# Ni nanocrystals on HOPG(0001): A scanning tunnelling microscope study

**DOI:** 10.3762/bjnano.4.48

**Published:** 2013-06-28

**Authors:** Michael Marz, Keisuke Sagisaka, Daisuke Fujita

**Affiliations:** 1 National Institute for Materials Science (NIMS), 1-2-1 Sengen, Tsukuba-city Ibaraki 305-0047, Japan; 2 Physikalisches Institut, Karlsruhe Institute for Technology (KIT), 76131 Karlsruhe, Germany

**Keywords:** clusters, growth mode, Ni, nickel

## Abstract

The growth mode of small Ni clusters evaporated in UHV on HOPG has been investigated by scanning tunnelling microscopy. The size, the size distribution, and the shape of the clusters have been evaluated for different evaporation conditions and annealing temperatures. The total coverage of the surface strongly depends on the evaporation rate and time, whereas the influence of these parameters is low on the cluster size. Subsequent stepwise annealing has been performed. This results in a reduction of the total amount of the Ni clusters accompanied by a decreasing in the overall coverage of the surface. The diameter of the clusters appears to be less influenced by the annealing than is their height. Besides this, the cluster shape is strongly influenced, changing to a quasi-hexagonal geometry after the first annealing step, indicating single-crystal formation. Finally, a reproducible methodology for picking up individual clusters is reported [1].

## Introduction

Metallic nanoparticles have been widely studied in the past few decades owing to their broad range of applications, such as catalysis [2–4], quantum dots [5] or chemical sensors [6]. Moreover, nano particles consisting of only some tens to a few hundred of atoms, so-called ’clusters’, have unique properties since they correspond to an intermediate system between isolated atoms and bulk. This makes clusters also suitable for fundamental studies of the crossover between the characteristics of single atoms and bulk properties [7–8]. Various methods are employed for growing metallic clusters on surfaces such as ion sputtering [9], pulsed laser deposition [10], electro deposition [11–13], vapor deposition [14], aerosol deposition [15], material transfer of an STM tip [16], etc. For the formation of nanoparticles a weak interaction of the metal with the substrate is favourable [17]. Graphite, in particular highly ordered pyrolytic graphite (HOPG), serves as an excellent substrate due to its low reactivity and large atomically flat surface. Among the transition metals the most commonly used materials for catalytic purposes are Ag, Pt, Pd, Cu, Rh and Ni [3]. Nickel clusters have recently received a lot of attention besides their catalytic properties [2–4] for the use as templates for the growth of small graphene islands [18].

The control of the size and the shape as well as the particle distribution play an important role for the aforementioned applications. Therefore, in this study we focus on the initial growing process of Ni clusters by evaporating Ni on the HOPG surface in ultrahigh vacuum (UHV). A quantitative characterization of the cluster properties, e.g., size, shape and distribution, for different experimental conditions has been performed by means of scanning tunnelling microscopy (STM) measurements. We show that Ni clusters are formed following a monomodal distribution, and that mild annealing can transform the Ni particles into single crystals. Within the annealing process two distinguishable regimes are observed. Additionally, we demonstrate the possibility to pick up single clusters from the surface in a controlled way, and we propose a model to understand the basics of the pick-up process.

## Experimental

All measurements were performed in an UHV-STM system from *Unisoku (USM-1200)* at liquid nitrogen temperature. The system consists of three separated chambers, including the load lock, preparation chamber and STM (analysis) chamber. The Ni deposition and annealing of the sample was carried out in the preparation chamber. The pressure during Ni deposition was usually below 6·10^−8^ Pa. The base pressure in the analysis chamber is presumed to be less than 3·10^−9^ Pa at 78 K. Electrochemically etched W tips and commercially available Pt/Ir tips (Unisoku) were used. The HOPG sample was mounted on a silicon strip that serves as heater for the annealing procedure. (Different HOPG crystals with different mosaic spread were used; however, we did not observe any influence of this in our data and refer to all of them by using the term HOPG substrate.) This setup allows local heating of the sample and avoids contamination from degassing of the surrounding stainless-steel parts. In order to get a clean surface the HOPG substrate was cleaved in conventional way with adhesive tape. After loading of the substrate to the UHV chamber, it was degassed at *T* ≈ 500 K for a few hours. The Ni-clusters were grown by evaporating Ni from a high purity Ni rod (5N purity) in situ by electron-beam heating. The deposition rate was controlled by monitoring the flux current. The flux ranged between 15 nA to 150 nA whereas the time was set between 30 s and 600 s. The total amount of deposited Ni is assumed to be proportional to the product of flux (*f*) and time (*t*) and the deposition coefficient (Π*_f_*_ × _*_t_*) is defined as Π*_f_*_ × _*_t_* = *f* × *t*. The resulting values of Π*_f_*_ × _*_t_* for the aforementioned variation of flux and time range from 1.5 μAs to 36 μAs. In the following the term “experiment” refers to a single value of Π*_f_*_ × _*_t_* with a specific combination of flux and time.

The HOPG substrate was cooled to liquid nitrogen temperature before the Ni deposition. For the Ni deposition, the substrate had to be transferred to the preparation chamber. Inevitably, the substrate warmed up (to quantify the temperature, measurements at ambient condition were performed; assuming a slower warm-up within the UHV chamber, the maximum substrate temperature was estimated to be lower than 200 K.), but we confirm that any influence of the substrate warmup on the clusters was not detected within the deposition times used in this study, cf. section “Influence of annealing on the clusters”.

A subsequent annealing process was performed in increments of approximately 100 K from 450 K up to 870 K, allowing a detailed study of the change of the clusters after each temperature step. The annealing temperature was monitored by optical pyrometers. The low annealing temperatures (*T* ≤ 550 K) were measured after the whole series of measurements in a second heating cycle, in which the same heating currents were used, which should result in a similar temperatures. Corresponding to this lack of accuracy, error bars of ±50 K have been plotted in the corresponding graphs. The STM measurements had to be performed at a relatively low scanning speed to prevent uncontrolled pick-up of the clusters, resulting in roughly 30 min scanning time per image.

Analysis of the STM data was performed by using the software *Gwyddion* (http://gwyddion.net/). In order to determine the cross sections parallel to the fast raster direction, raw data were used. The cluster dimensions were determined by fitting these cross sections with a rectangular function. After fitting a large number (79–150) of clusters randomly selected from several scanning areas for each experiment, height and width histograms were plotted (not shown). The resulting histograms showed a monomodal normal distribution for the diameter and the height of the clusters. To determine the mean values for each experiment, the histograms were fitted with Gaussian curves, and details are given below. The relative coverage of the surface and the number of clusters were extracted by implementing the appropriate options in the software. After subtracting the mean plane and setting the lowest value to zero, the clusters were marked by choosing an appropriate threshold value for *z* above the substrate. The mean value of the height and width, and the number of clusters were calculated from these data.

## Results and Discussion

### Growing behaviour

**Figure 1:**
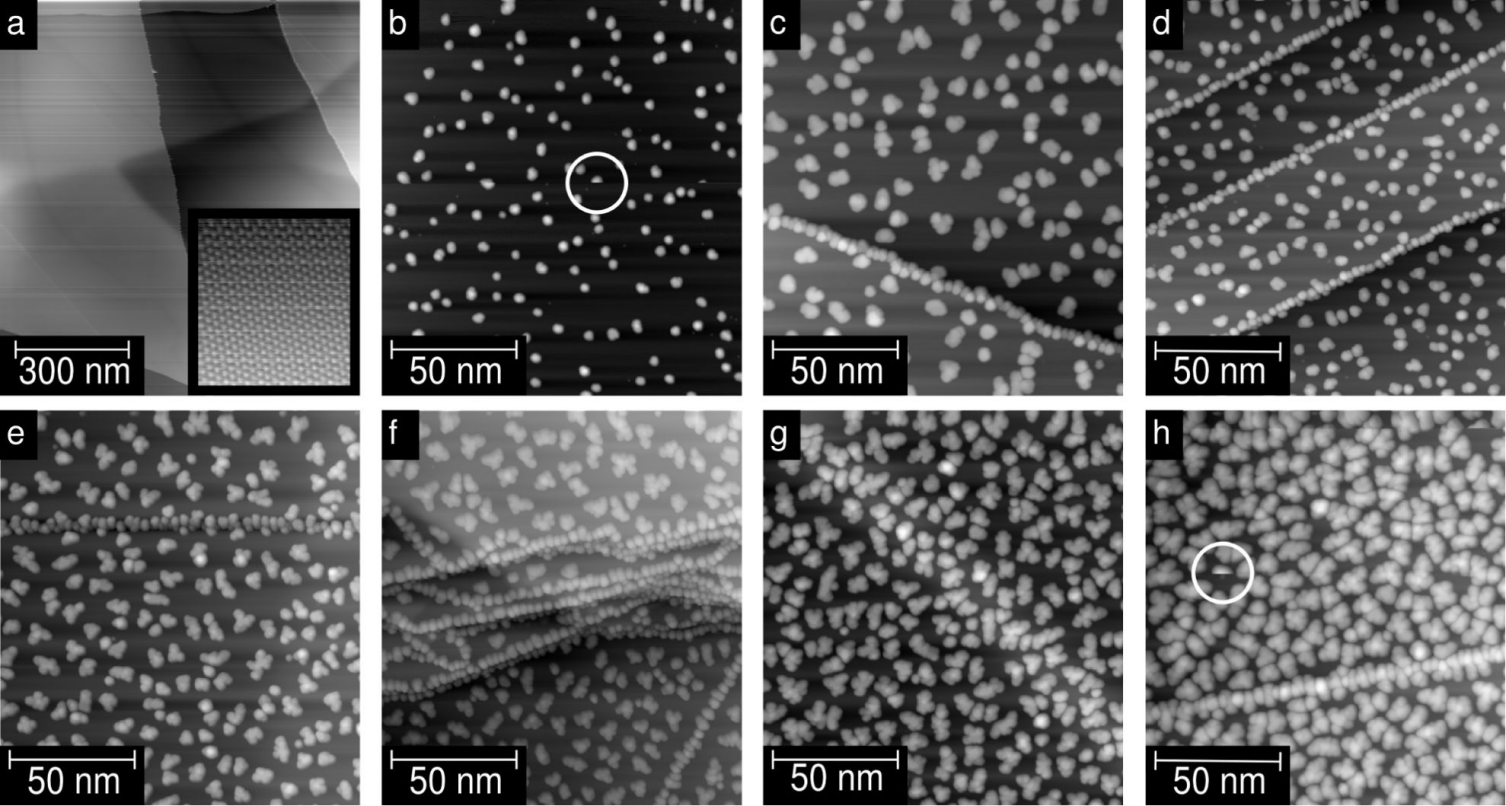
Evaporation time and flux dependence of the growth of the Ni-clusters without further annealing. STM topographic images of (a) freshly cleaved HOPG. The image shows a large-scale scan (*V* = 2 V; *I* = 0.2 nA), the inset shows atomic resolution (*V* = 0.3 V; *I* = 0.5 nA). (b–h) Evaporated Ni clusters on HOPG (*V* = 1 V; *I* = 0.2 nA). Evaporation parameters: (b) flux ≈50 nA, time = 30 s → Π*_f_*_ × _*_t_* = 1.5 μAs; (c) ≈15 nA, 300 s, 4.5 μAs; (d) ≈100 nA, 50 s, 5.0 μAs; (e) ≈50 nA, 180 s, 9.0 μAs; (f) ≈100 nA, 100 s, 10.0 μAs; (g) ≈145 nA, 100 s, 14.5 μAs; (h) ≈150 nA, 160 s, 24.0 μAs.

Figure 1 gives an overview of the obtained Ni clusters under various evaporation conditions without any annealing; the deposition parameters are given in the figure caption. The sequence of images in Figure 1 is ordered from low to high density of Ni particles. The cluster density increases by increasing Π*_f_*_ × _*_t_*. Figure 1a displays the freshly cleaved and degassed HOPG surface for reference. The width of the terraces was several hundreds of nanometers. The inset of Figure 1a depicts a typical image of atomically resolved HOPG. Figure 1b–Figure 1h show that the clusters form cloud-like shapes on the HOPG surface. For the nonannealed samples, the Ni-clusters appear to be randomly distributed on terraces but tend to grow preferentially along step edges (Figure 1c–Figure 1f and Figure 1h) in agreement with previous STM investigations [17,19–20]. Yang and Sacher have shown by artificially introducing defects by Ar sputtering, that defects act as nucleation centres for Ni on HOPG [20]. In our case the sticking coefficient of Ni on the basal plane for low temperatures seems to be large enough to promote the nucleation on the basal plane even without additional defects. With increasing coverage, the clusters on the terraces tend to stick together in random directions, forming conglomerates with random shapes. This clustering of the Ni clusters makes it difficult to define the number of clusters on the surface. In contrast to this, the clusters at the step edges agglomerate preferentially along the step edge, indicating a larger step–cluster interaction than cluster–cluster interaction. On the other hand, subsurface defects seem to have less influence on the clusters, as is visible in Figure 1g. This finding is consistent with the observation that the mosaic spread does not influence the cluster growth, since the mosaic spread results also from the mismatch of subsurface layers.

**Figure 2:**
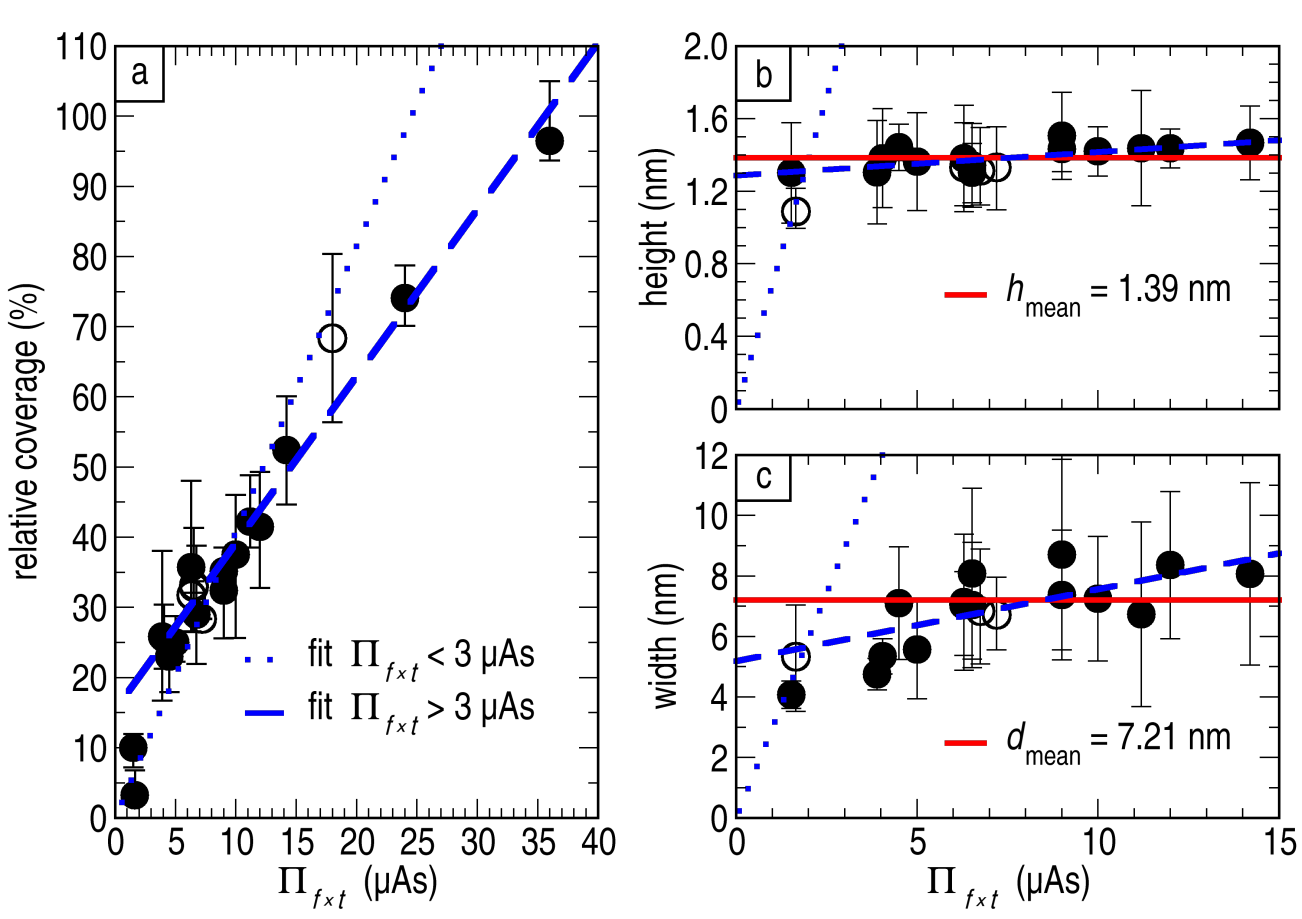
Analysis of the growing behaviour: (a) coverage of the surface, (b) cluster height and (c) cluster width versus Π*_f_*_ × _*_t_*. Panel (b) and (c) have the same scale on the *x*-axis. Plotted as the filled symbols are the results obtained by fitting cross sections, as described in the text. The error bars indicate the ±2σ confidence level. Additional data points are plotted with empty symbols in the graphs to point out the lack of statistics (less than 25 cross sections), accordingly the arithmetic mean value was calculated. The error bars indicate the largest and smallest value obtained from the fit of the individual clusters. For high coverage (Π*_f_*_ × _*_t_* ≥ 15 μAs) the cluster–cluster distance was too small for getting reliable values. Thus, we do not have data for the height and the width for these experiments.

The overall size of the clusters is determined by the interplay of the adhesion of Ni on the clean surface and the Ni–Ni interaction, which is typical for a Vollmer–Weber growth. For a detailed statistical analysis of the results, values such as the mean cluster height and width have been calculated. Figure 2a shows the dependence of the surface coverage with Ni-clusters in percentage with respect to the total amount of evaporated Ni (Π*_f_*_ × _*_t_*). From a linear fit, we can conclude that the HOPG surface is fully covered by clusters when the deposition of Ni reaches Π*_f_*_ × _*_t_* = 36 ± 2 μAs. For the calculation, the values corresponding to a very low total amount of Ni, i.e., Π*_f_*_ × _*_t_* ≤ 3 μAs (dashed blue line), have been excluded, and we will comment on this later.

Figure 2b shows that an almost constant height of the cluster, independent of any evaporation condition, is obtained. The calculated mean value of the height is *h*_mean_= 1.39 ± 0.06 nm (red solid line), which corresponds to at least four times the bulk lattice constant of Ni. However, a linear growth cannot be excluded from our data. Using a linear fit for the data (dashed blue line) results in a line with a very small slope. The calculated maximum height for one monolayer coverage with clusters in this case is expected to be *h* = 1.70 nm, i.e., five times the bulk lattice constant of Ni. Using this result, we can estimate the deposition rate as a function of the flux to be 1 μA, which corresponds to 0.038 nm/s. A similar behavior is observed in Figure 2c for the width of the clusters. The mean value of the width obtained from these data is *d*_mean_ = 7.21 ± 1.30 nm (red solid line). If we perform a linear fit (dashed blue line), this also results in a line with small slope. For higher coverage, the clusters tend to agglomerate making the determination of individual clusters hard, and therefore, the determination of a meaningful width for a single cluster is difficult.

We now comment on the relatively large deviation from the mean value for small Π*_f_*_ × _*_t_* ≤ 3 μAs in the coverage and the height. We presume that in the very initial state of the nanoparticle growth, the clusters cannot reach their maximum height and width due to an insufficient amount of Ni. Within this assumption, a linear function was drawn in all panels of Figure 2 (dotted blue line) to reveal a different growth behaviour with a fast increase at the very early stage. This matches well with the expected Volmer–Weber growth as initial growth state for Ni on HOPG. For the deviation of the data for low Π*_f_*_ × _*_t_* from the linear behaviour of the coverage the same argument is valid. The clusters grow first to their maximum height and then new clusters are formed on the substrate. Therefore, the coverage will be significantly lower for very low Π*_f_*_ × _*_t_* as no new clusters are formed. Another possibility for the deviations in Figure 2 could be a significantly lower sticking coefficient when only a small amount of Ni is deposited. In this case our assumption of the proportionality of Π*_f_*_ × _*_t_* to the total amount of deposited Ni would be no longer valid.

**Figure 3:**
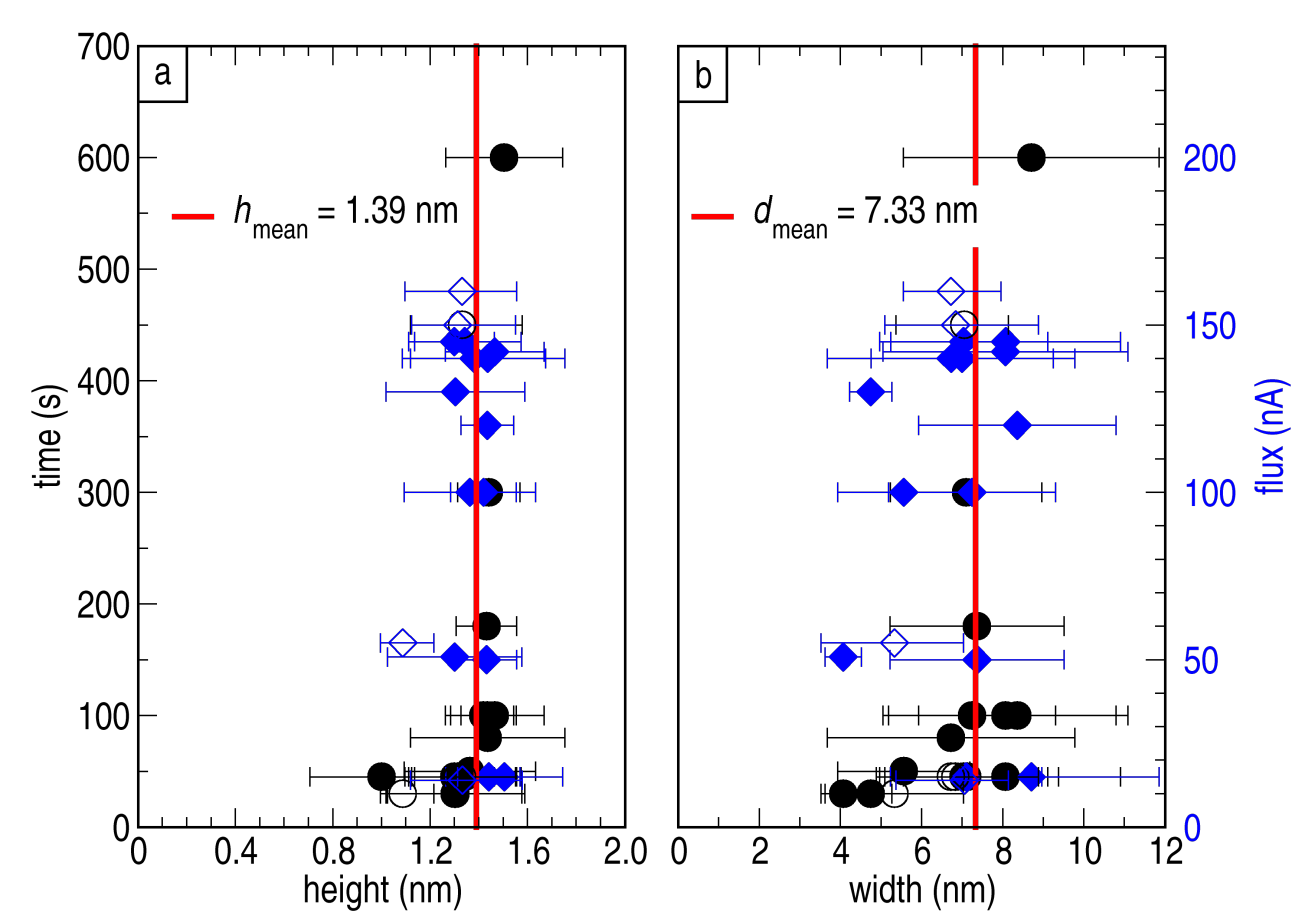
Analysis of the height (a) and width (b) dependence on deposition time (black circles) and flux (blue diamonds) during the evaporation process. The plots show no significant influence of the evaporation conditions on the size of the clusters.

We have demonstrated that the size of the clusters does not depend on the evaporation condition. In order to support our claim, we plot deposition time (black circles) and rate (blue diamonds) versus height and width in Figure 3a and Figure 3b, respectively. The graphs also include the aforementioned values of the mean height and the mean width (red solid lines). Within these plots no significant dependency of the height and width on the time or the flux is found. Moreover, we do not observe any dependency in height and width by adequately varying flux and deposition time to obtain similar Π*_f_*_ × _*_t_* (not shown). Out of these plots, we draw two important conclusions. First, the cluster height and width can be considered independent of the evaporation conditions when the coverage of clusters is below one monolayer. Second, resulting from the first observation, the warm-up of the substrate during the deposition is negligible. The cluster diameter in the presented work is constant and roughly one order of magnitude smaller than for nickel deposition at room temperature, as done for example in the work of Bastl et al. [19]. Therefore we conclude that the temperature for the whole deposition process was significantly lower than room temperature.

### Influence of annealing on the clusters

**Figure 4:**
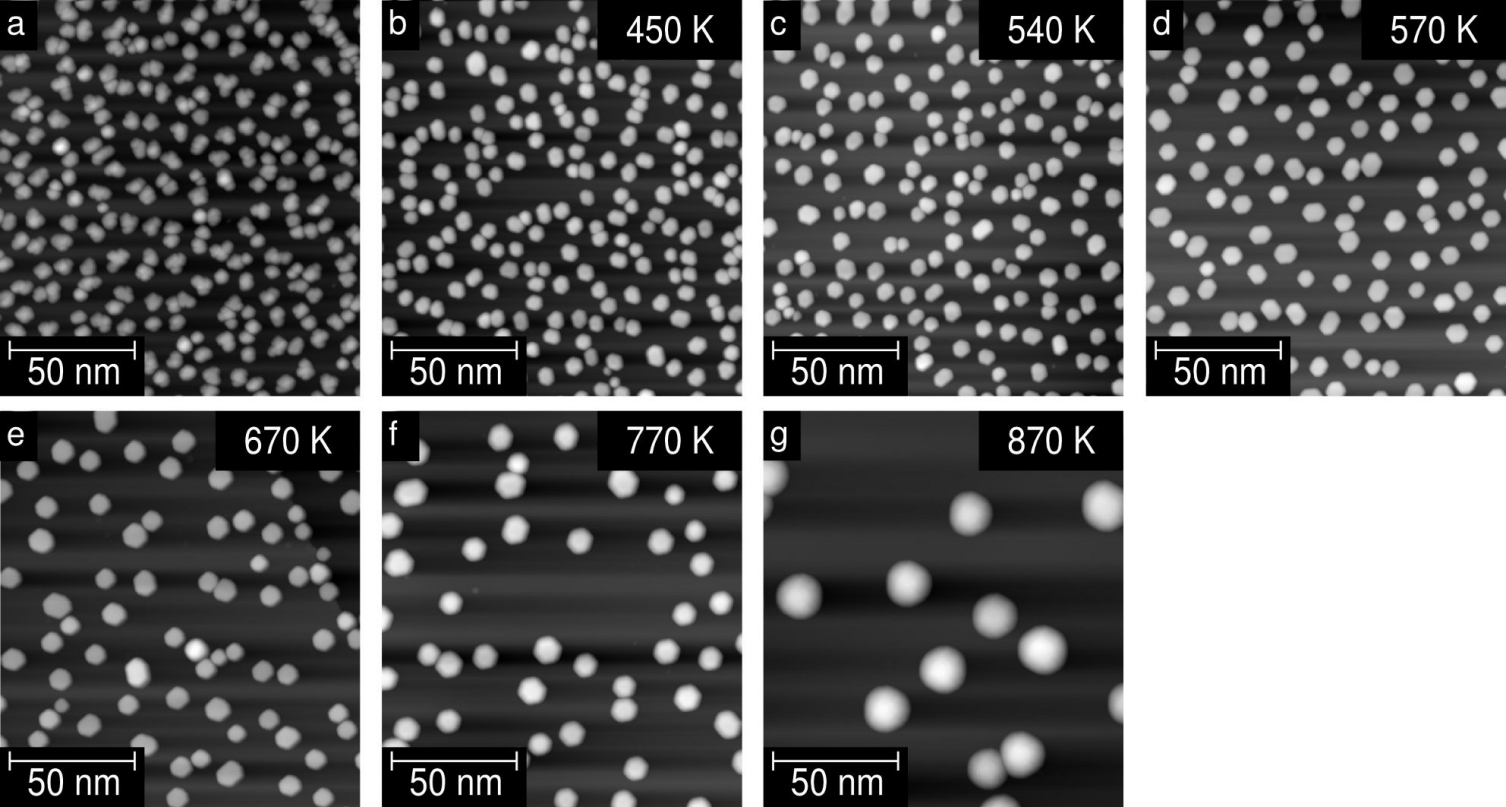
Temperature dependence of the annealing behaviour of the Ni clusters. STM topographic images of the HOPG sample (a) after deposition of Ni on the cold sample and (b–g) after the different annealing steps. Annealing was performed stepwise, starting at *T* ≈ 450 K (b) up to *T* ≈ 870 K (g). All data were obtained at *V* = 1.0 V with a tunnelling current of *I* = 0.2 nA in consistency with Figure 1.

Figure 4a was recorded directly after depositing Ni on the cold surface without annealing. As previously discussed, the clusters appear randomly distributed, and bear cloud-like shapes. Thus, we assume the clusters not to exhibit single crystallinity. Figure 4b–Figure 4g show STM images of the surface after a stepwise annealing process from 450 K to 870 K. Figure 4b shows the sample after annealing for one hour with constant current and a maximum temperature of *T*_max_ = 450 K. It is noticeable that the cluster shape has changed drastically. Now the Ni-clusters exhibit a quasi-hexagonal structure, suggesting the formation of single crystals due to the mild annealing. For higher annealing temperatures the hexagonal structure remains visible, see Figure 4c–Figure 4f. However, for the highest used annealing temperature (*T* ≈ 873 K) the hexagonal shape can no longer be resolved. In this case the height of the clusters is almost doubled compared to the one produced by the annealing at *T* ≈ 773 K. Note that for such a height of the clusters the tip geometry will play an important role and is most likely the cause of the change in the observed width and shape. Also jumps of one or more clusters to the tip may have changed its resolution. Nevertheless, the change in lateral resolution has no impact on the determination of the height of the cluster and for counting the number of clusters on the surface. We notice that the height is more affected than the width of the cluster by the annealing process.

**Figure 5:**
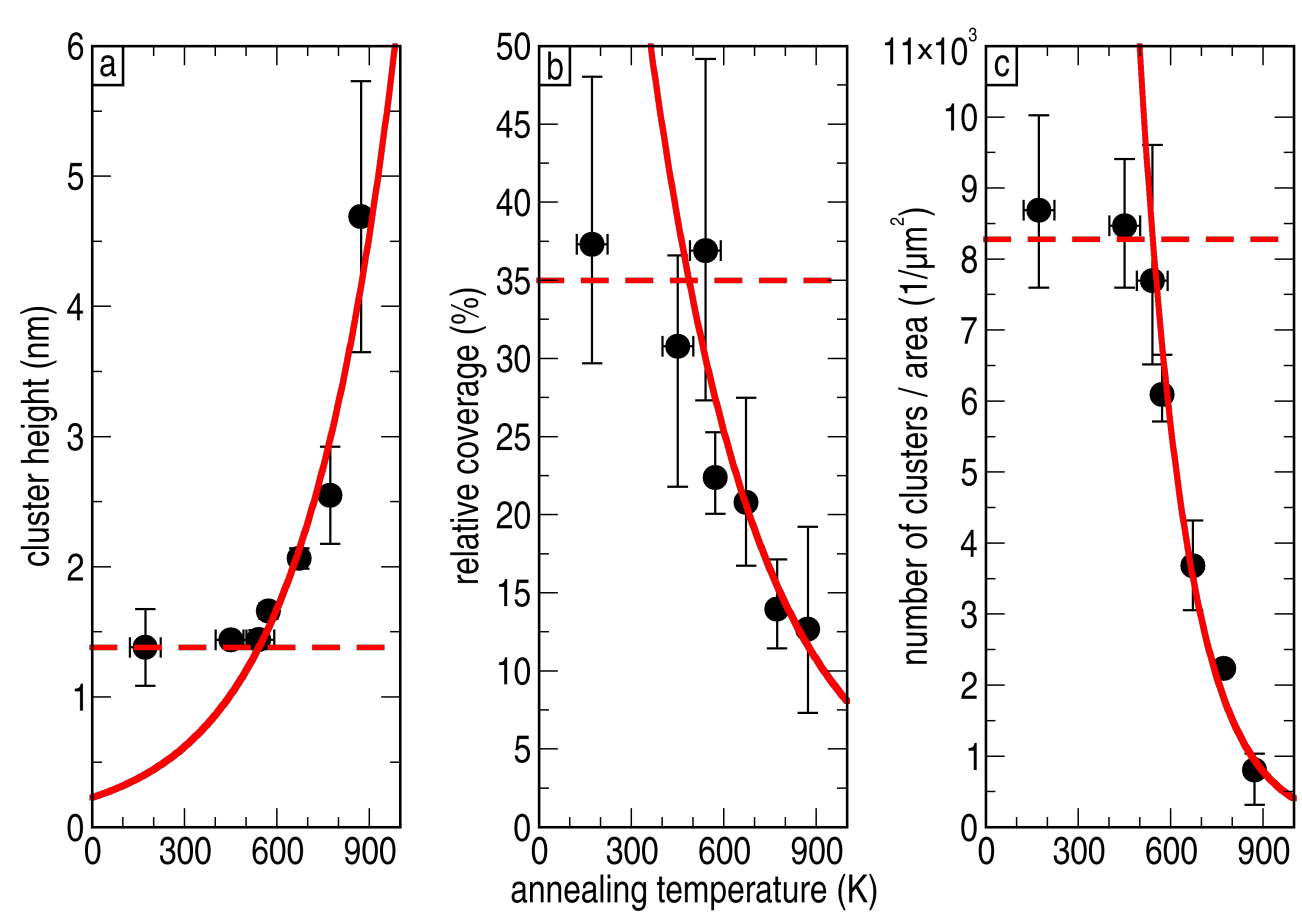
Temperature dependence of the annealing behaviour for (a) the height of the clusters, (b) the percentage of surface covered by the clusters, and (c) the number of clusters per area (cluster density *n*). The annealing process can be divided into two parts: First, for mild annealing (*T* ≤ 500 K) where no diffusion takes place (indicated by the red dashed line), and second, for higher annealing temperatures with an exponential increase in height, and an exponential decay in coverage and number of clusters per area (solid red line).

To discuss the effect of annealing on the number of cluster and their sizes, a sample with a medium coverage deposited at high flux has been used. At medium coverage, there is a large number of clusters, but it is still possible to measure their height, width and number. Figure 5a–Figure 5c shows the temperature dependence of the cluster height, the relative coverage of the surface and the number of clusters per area, respectively.

To provide a more physical insight into these results, we will make the following considerations. After annealing, the sample is transferred back to the STM chamber where it is cooled down for performing the measurements. The diffusion is allowed only at elevated temperatures during the annealing process. Therefore, the STM measurements represent a snapshot of the diffusion process that took place at the last annealing temperature. Following this assumption the diffusion energy *E*_d_ can be determined. Starting from the density of clusters *n*, the mean distance between two clusters *L* can be calculated, assuming an equidistant separation between them, according to

[1]
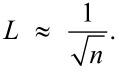


**Figure 6:**
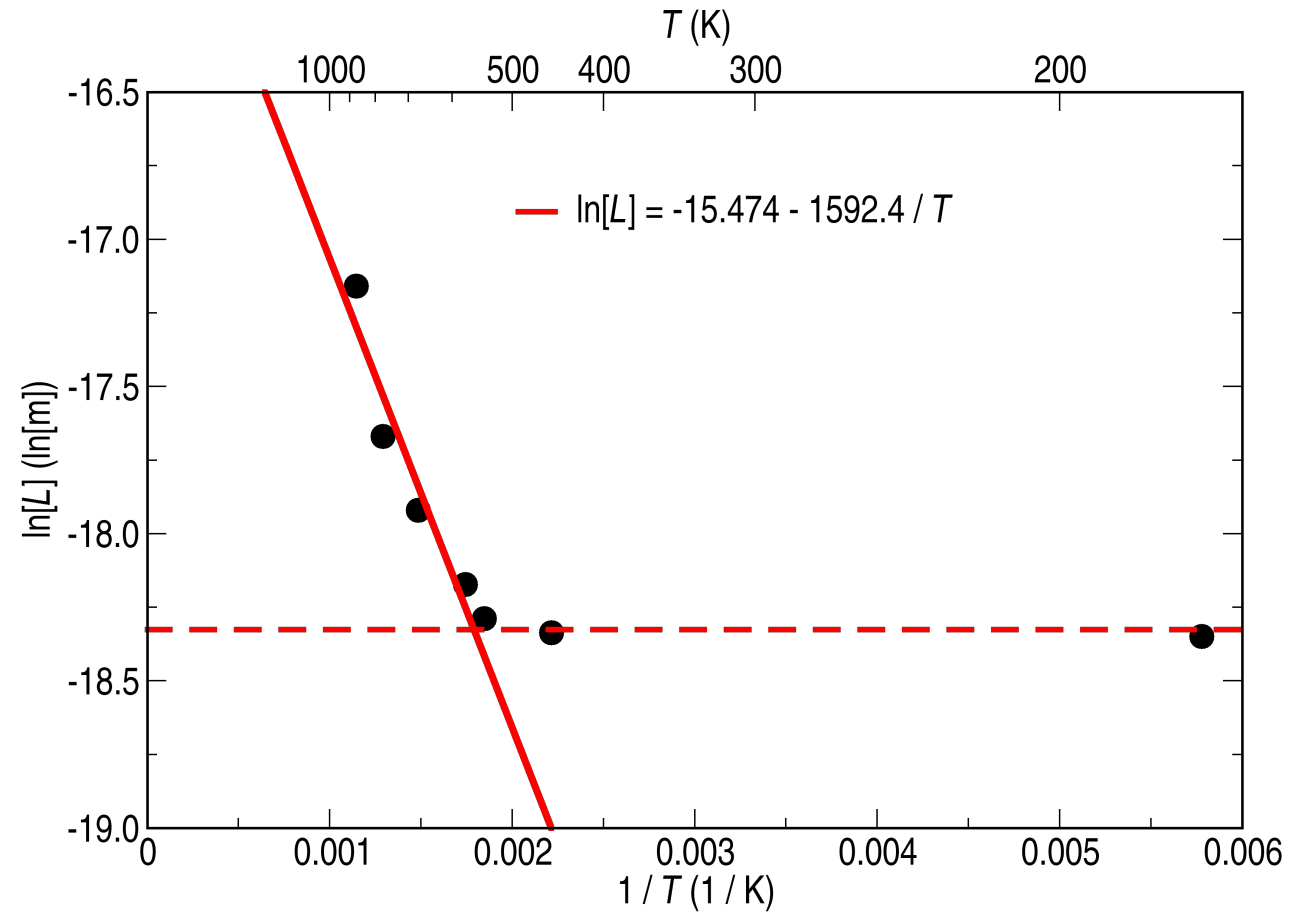
Arrhenius plot for analyzing the diffusion process of the Ni on HOPG. Two regimes are distinguishable in the graph and are emphasized with two linear fits to the data.

We consider this length *L* as the mean diffusion length of the clusters. The diffusion energy *E*_d_ can then be determined by an Arrhenius plot, as depicted in Figure 6, following [21], being

[2]
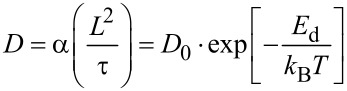


[3]
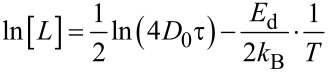


where *τ* indicates the time between two jumping events, and *α* is 1/4 assuming a two-dimensional diffusion. Figure 6 can be divided into two different regions that can be interpreted as two distinct diffusive regimes. First, in the low temperature regime, the data points lay on a constant curve (dashed line in Figure 6) indicating that any diffusion hardly takes place. Second, in the high annealing temperature regime, we fit a linear regression to the data (solid line in Figure 6) which results in a diffusion energy of *E*_d_ = 0.274 eV (*E*_d_ = 4.40·10^−20^ J). This energy is in good agreement with diffusion energies for Au on HOPG [22] (*E*_d_ ≤ 0.3 eV) and Ni in graphite [23] (*E*_d_ = 0.807 eV), noting that the diffusion barrier at the surface is smaller than in the bulk.

These two regimes are also evident in the dependency of the number of clusters, cluster size and total coverage on the annealing temperature. The three graphs of Figure 5 show that in the low-temperature annealing range their value is almost constant, and that only the crystallinity changed due to the change in atomic arrangement within the clusters. At higher annealing temperatures, however, the number of clusters is reduced, whereas their height is increased significantly. This agrees well with the idea of Ni-atom or rather Ni-cluster diffusion. According to this interpretation, two fits are presented in the panels (a)–(c) of Figure 5. The dashed lines in all panels represent the regime where no Ni diffusion is observed. In the case of the height, panel (a), the regime of no diffusion equals the mean value obtained from the nonannealed samples. The solid lines are obtained by fitting an exponential increase for the height and an exponential decay for the coverage and number of clusters to the data. The exponential decay of the coverage results directly from the exponential increase of the distance between the clusters, as shown by the fit in Figure 6, when the diameter of the clusters stays constant. The same argument is valid for interpreting the exponential increase in the cluster height, if we assume that neither evaporation from the surface nor diffusion into the substrate of the Ni clusters occurs during heating of the sample.

We have shown that annealing of the cluster can be separated into two regimes. First, single-crystalline clusters are formed. Second, with further annealing the single-crystalline clusters mainly grow in height whereas their width seems to be less affected. These two regions are also distinguishable within a simple analysis of the diffusion process.

Concluding the discussion about the growth mode and annealing behaviour of the Ni-clusters, we want to comment on the possibility of carbon interdiffusion into the clusters. Sinharoy and Levenson showed the formation and decomposition of Ni_3_C in Ni-films deposited on HOPG [24]. Additionally, it is also known from Monte Carlo simulations by Diarra et al. that the solubility of carbon in Ni is significantly increased for small clusters [25]. Although we cannot exclude completely the diffusion of carbon into the clusters and the formation of nickel carbide, we have reasons to believe that it is negligible in our experiments. First, the deposition was done at low temperatures at which we expect the carbon mobility to be much lower than at room temperature. Second, the solubility of carbon in clusters of approximately 1000 atoms is already comparable to the solubility in the bulk, which is only 5–6% [25]. Finally, Ni_3_C decomposes at relatively low temperatures, i.e., ≈670 K [24,26], which is below some of our annealing temperatures. However, we do not observe any significant change in the clusters, although our annealing experiments made a crossover from below to above this temperature.

### Pick-up of individual clusters

**Figure 7:**
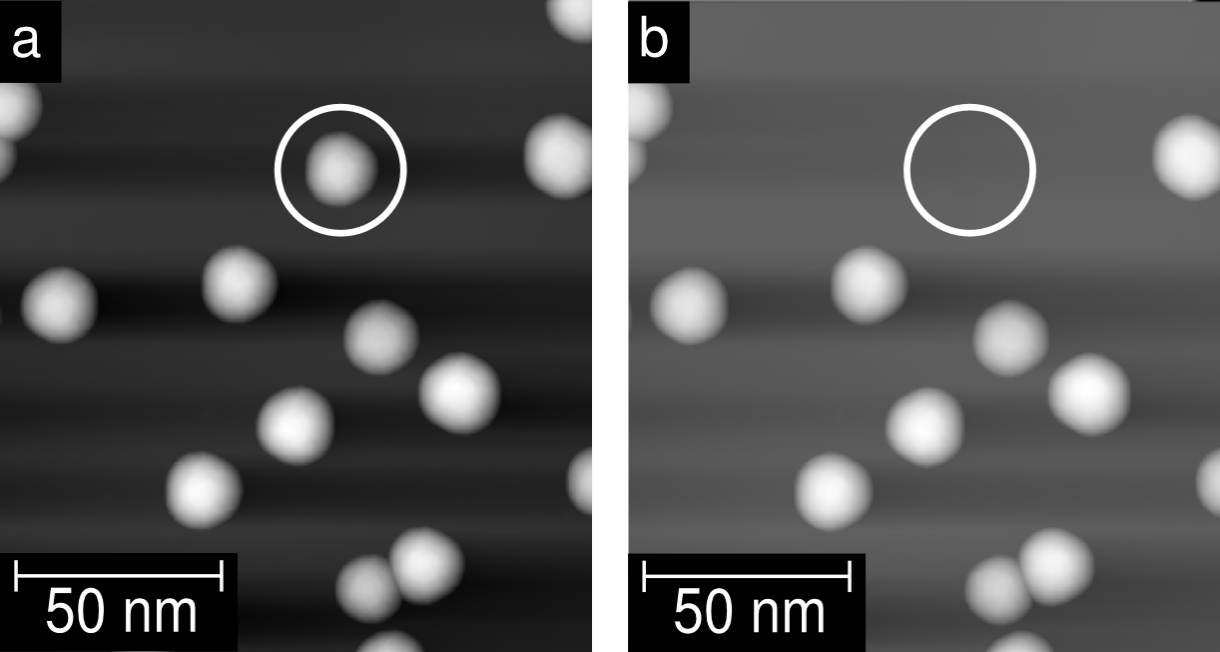
STM images of the HOPG surface (a) before, and (b) after the picking up of an individual cluster. The area of interest is marked with a white circle.

The Ni-clusters are only loosely bound to the surface, and thus, it is possible to pick up individual clusters with the STM tip in a controlled way. One demonstration of the pick-up is given in Figure 7 with topographic STM images before (a) and after (b) the picking up of the cluster.

**Figure 8:**
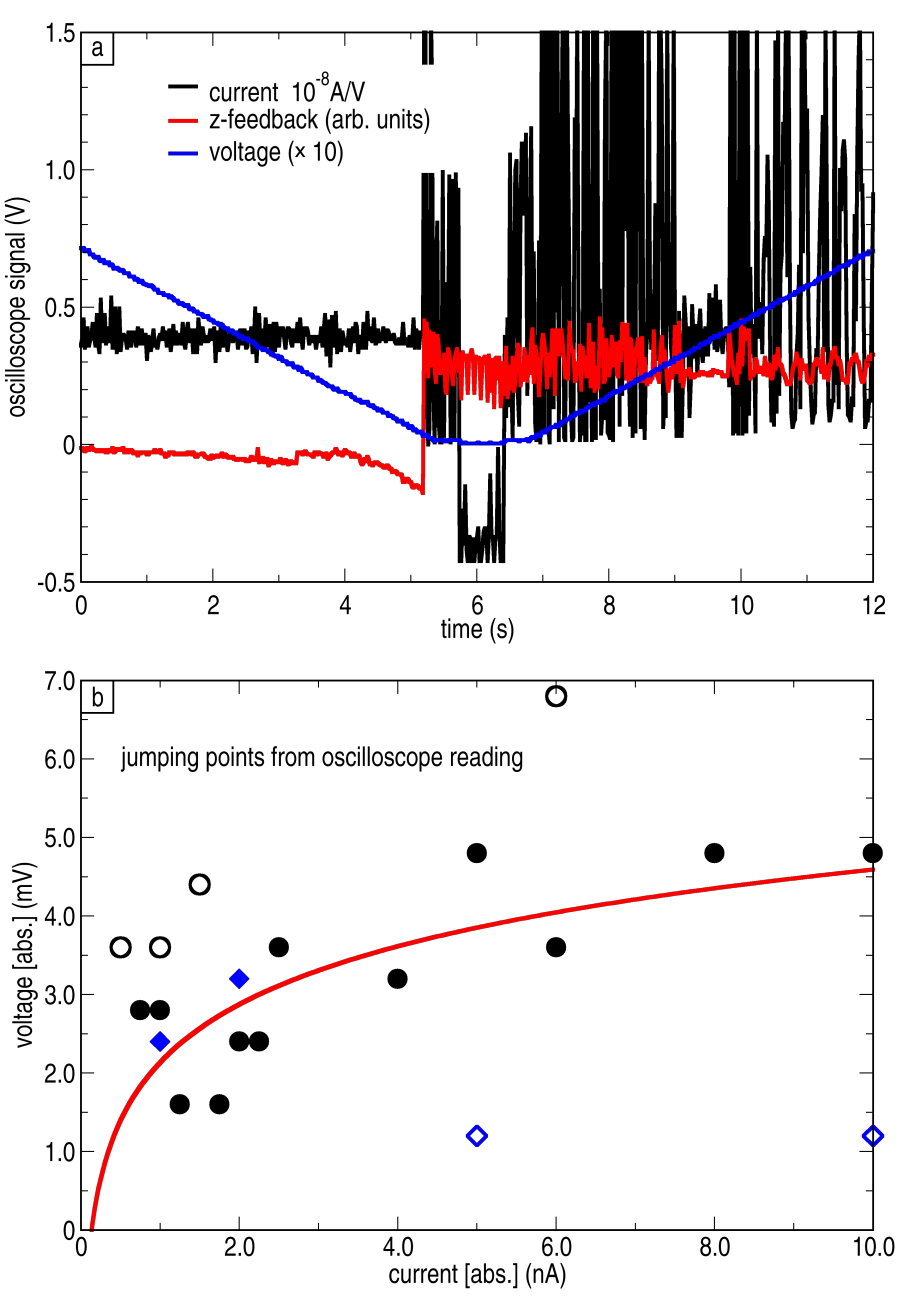
(a) Typical records of the current, bias and *z*-feedback signals for a successful pick-up. The jumping voltage is read out at the point where the first instability in the current or *z*-feedback signal is observed. (b) Pick-up voltage for different current setpoints. Bias ramp was started at +1 V (circles) or −1 V (diamonds). The data of the filled symbols were used for fitting the exponential curve (solid line).

The basic idea of the process is based on gradually reducing the cluster–tip distance, until the attractive force between tip and cluster overcomes the adhesive force of the Ni nanocrystal on the HOPG surface. For this purpose, the tip was first stabilized above the center of the targeted cluster, and then the current setpoint was increased to a value up to 10 nA while the z-feedback was kept enabled. Since the tunnelling current depends, in a first-order approximation, exponentially on the distance, an increase of one order of magnitude in the current results only in about one ångström decrease in the cluster–tip distance. In order to further decrease the distance, the bias (a 1/10 divider was installed to enhance the resolution) was gradually decreased with closed *z*-feedback loop. Since we used small bias values, we can assume that the distance depends linearly on the voltage. As soon as a jump in the current or *z*-feedback signal was observed, the bias was gradually increased again. Afterwards, the usual tunnelling conditions were readjusted (*V* = 1.0 V, *I* = 0.2 nA), and the outcome of the pick-up attempt was checked with a topographic scan. In roughly 50% of the attempts the pick-up was successful. We point out that an instability in the *z*-feedback and current signal is always observed in a successful pick-up attempt. The current, bias and *z*-feedback signals measured for a successful pick-up are given in Figure 8a. In this case, the current was set to *I* = 4 nA and the instability occurred at *V* = 3.2 mV.

Figure 8a shows that immediately after the pick-up the system becomes unstable for a relative long time. We ascribe this behaviour to instabilities at the tip, for example through the rotation or break-up of the cluster. After increasing the bias and resetting the current setpoint to the original value, the feedback-loop was usually stable again, but the resolution of the tip was often worse. Furthermore, the probability for an unintended pick-up of a second or more clusters was enhanced during topographic scans after a successful pick-up. Figure 8b reveals the correlation between the current set-point and the voltage where the pick-up happened for about twenty successful attempts.

Despite the large scattering of the data, an exponential dependence of the jumping voltage on the current set-point can be observed and the data was fitted by an exponential behaviour (solid line). For fit the data points with large deviation (open symbols) have been neglected. The result reflects the common exponential dependence of the current set-point on the tip–sample distance, when we assume that the distance decreases proportionally with the applied bias. This observation supports that mainly the tip–cluster distance is important for the pick-up process and not the applied bias, i.e., the applied electric field.

We will now take a closer look at the forces and interactions involved in the process. As mentioned before, the adhesive energy has to be overcome to remove the cluster from the surface. We can estimate the necessary force for this process in the DMT limit (Derjaguin–Muller–Toporov theory [27]), i.e., in the limit of small deformations. Thus, the adhesive force can be written as

[4]
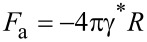


[5]
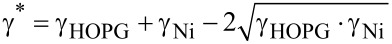


where *γ*_x_ is the surface tension and *R* the radius of the cluster, assuming a spherical particle. With *γ*_HOPG_ = 1.75 J/m^2^, *γ*_Ni_ = 2.45 J/m^2^, and *R* = 5.9 nm, a pull off force of *F*_a_ = −4.36 nN is expected. The attractive force between tip and cluster can be divided into three different contributions: Dielectrophoretic force, image force and van der Waals force [15,28].

[6]
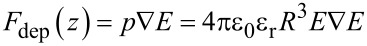


[7]
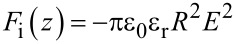


[8]
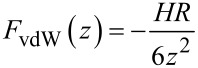


where the electric field *E* = *E*(*z*) is a function of the tip particle distance *z*. Since our experiments were performed in UHV, and no water interlayer is present, the Hamaker constant can be considered to be *H* = 4·10^−19^ J, and ε_r_ = 1 is valid. For simplicity, the electrical field is considered not to be influenced by the presence of the clusters and its value to be constant over the whole width of the cluster, leading to the convenient form *E*(*z*) = *V*/*z*. Furthermore, a good conductivity between cluster and substrate is assumed leading to the disappearance of the dipole moment *p* and consequently the dielectrophoretic force. Taking these assumptions into account, the total attractive force can be written as:

[9]
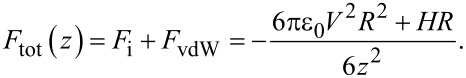


Noticing that in our experiments the bias at which the pick-up takes place is quite small, we can conclude that the whole process is dominated by the van der Waals interaction:

[10]
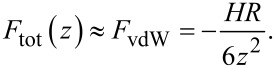


**Figure 9:**
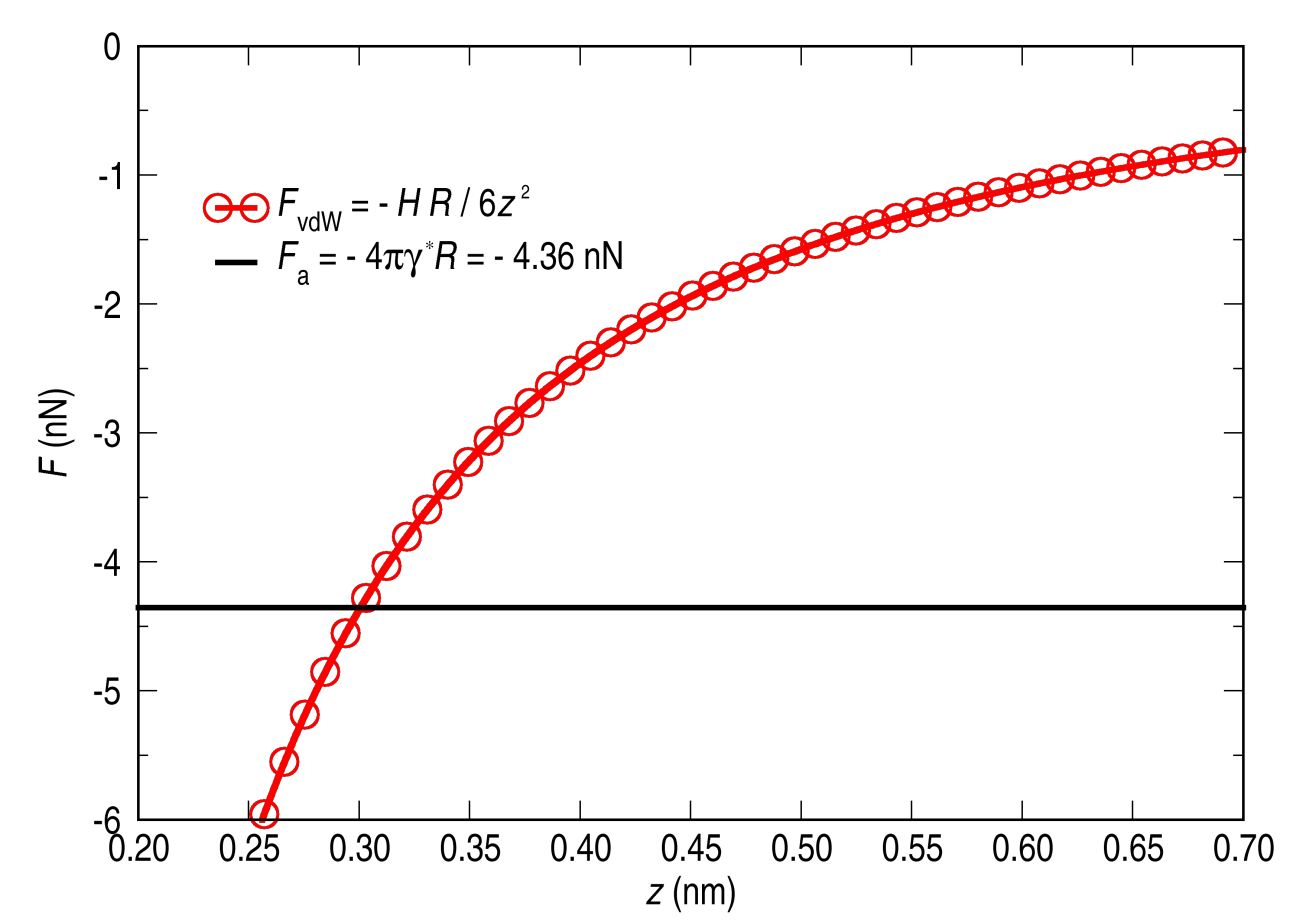
Forces involved in the process of picking up a Ni cluster. The tip–sample distance at which the attractive force overcomes the adhesive one is estimated to be 0.3 nm.

In Figure 9 the dominant forces involved in the pick-up process are plotted: the adhesive force with a constant value of *F*_a_ = −4.36 nN (solid black line) and the distance-dependent van der Waals force (open red circles). This plot provides a graphical determination of the tip–sample distance at which, following our model, the pick-up occurs. The obtained tip–cluster distance is *z* = 0.30 nm. This value is in a reasonable range taking into account the applied tunnelling conditions. Besides the controlled pick-up of the cluster, jumps of clusters to the tip occur frequently during the scan.

One example is marked with a white circle in Figure 1b and Figure 1h, respectively. The jumping rate is more enhanced when a higher tunnelling current or a faster scanning speed is used during a scan. In the case that a higher tunnelling current is used, a smaller tip–cluster distance is the reason for the rise of the jumping rate. In the case that a faster scanning speed is used, the *z*-feedback time constant is not fast enough to react to the steep increase of the height at the cluster edges resulting in a small tip–cluster distance. So far we have not succeeded in reversing the process, i.e., once a cluster is picked up it remains bound strongly to the tip.

## Conclusion

In this study, the growth mode of small nickel metal clusters on HOPG has been investigated by scanning tunnelling microscopy. Statistical analysis of the STM images indicates that the total number of Ni clusters and the relative coverage depend linearly on the total amount of deposited Ni. The height and width of the cluster are independent of the deposition conditions, in particular of the substrate temperature (for temperatures estimated to be between 100 K and 200 K), deposition time and rate. After the annealing process, the coverage of the surface is reduced with increasing annealing temperature. Mild annealing slightly above room temperature already results in a change of the cluster shape, from a cloud-like to a quasi-hexagonal structure, indicating single-crystal formation. The lateral size of the clusters seems to be hardly affected by the annealing. In contrast to this, the cluster height continuously increases with annealing temperature. After deeply analysing the data we found that two diffusion regimes can be distinguished during the annealing process. For mild annealing temperatures (*T* ≤ 500 K) only reorganization of the atoms within the clusters takes place, whereas for higher annealing temperatures (*T* ≥ 500 K) Ni-atoms and even whole Ni-clusters diffuse on the HOPG surface. Finally, controlled picking-up of individual Ni clusters with the STM tip has been described.
